# Development and implementation of an electronic health record system for use in humanitarian emergencies, disaster response, and conflict zones

**DOI:** 10.1371/journal.pgph.0003124

**Published:** 2025-01-29

**Authors:** Sarah D. Draugelis, Erik C. Brown, Donald A. Donahue, Justin Hickman, Sean G. Smith, Philip Sutherland, George A. Yendewa, Amir M. Mohareb

**Affiliations:** 1 Team fEMR, St. Clair Shores, Michigan, United States of America; 2 Department of Neurosurgery, Valley Children’s Hospital, Madera, California, United States of America; 3 University of Maryland Baltimore, Baltimore, Maryland, United States of America; 4 Beth Israel Deaconess Medical Center, Boston, Massachusetts, United States of America; 5 Critical-Care Professionals International, Graham, Florida, United States of America; 6 Department of Medicine, Case Western Reserve University, Cleveland, Ohio, United States of America; 7 Division of Infectious Diseases and HIV Medicine, University Hospitals Cleveland Medical Center, Cleveland, Ohio, United States of America; 8 Center for Global Health, Massachusetts General Hospital, Boston, Massachusetts, United States of America; 9 Division of Infectious Diseases, Massachusetts General Hospital, Boston, Massachusetts, United States of America; 10 Department of Medicine, Harvard Medical School, Boston, Massachusetts, United States of America; PLOS: Public Library of Science, UNITED STATES OF AMERICA

## Abstract

Humanitarian medical response to natural and human-made disasters can be complicated by high clinician, staff, and patient turnover. While electronic medical records are being scaled up globally, their use remains limited in humanitarian response settings. The Fast Electronic Medical Record (fEMR) system is an open-source electronic health record system specifically designed for use in resource-limited settings and humanitarian crises. The system was developed between 2010–2014 through an iterative design process with multidisciplinary team members. It was operationalized in settings with and without internet connectivity. We analyzed data on fEMR usage since inception until October 2022 to estimate the number of patients served by the system. In eight years of implementation (2014–2022), the fEMR system has been deployed 60 times to 11 different countries across four different continents by 14 different organizations. These deployments collectively account for over 37,500 patient encounters with an estimated 31,940 distinct patients. The settings of fEMR use ranged from refugee and migrant health clinics near the Mexico-US border to the Poland-Ukraine border in the context of the 2022 war in Ukraine. User feedback demonstrated the program’s ease of use by providers of different clinical and technical backgrounds. Feedback primarily emphasized improving the system’s hardware requirements and workflow. The simple design allowed for clinician users to adapt the system to a variety of clinical scenarios. Ongoing and future work in adapting electronic health records to international humanitarian response will emphasize data security, patient privacy, equity, and the rapid translation of electronic health data to improve population health. In humanitarian response settings, electronic health records can improve quality of care and provide a source of clinical and management data for public health planning.

## Introduction

More than 274 million people around the world are impacted by complex humanitarian emergencies [1]. Delivery of medical care in resource-limited settings is greatly complicated by the presence of natural and human-made disasters. These include events such as war, political unrest, and extreme weather events, which are frequently associated with forced displacement [1]. There are more than 100 million people in the world classified as refugees (i.e., fleeing their countries due to a well-founded fear of persecution), asylum seekers, or internally displaced persons based on estimates from the United Nations [2]. In such circumstances, medical care is frequently oriented toward providing emergency assistance and preventing large-scale suffering, for example, by preventing outbreaks of infectious diseases [1]. Limitations in the availability of life-saving equipment, medications, personnel, and access to treatment centers worsen the morbidity and mortality associated with humanitarian emergencies [3].

Providing medical care in humanitarian emergencies requires overcoming numerous logistical challenges, including unexpected resource constraints, the transient nature of clinicians, support staff, and patients, and the absence of individual historical healthcare data. Following a humanitarian disaster, victims of forced migration frequently seek care between different clinics, hospitals, pharmacies, and temporary, often ad hoc, treatment centers [4–6]. The fragmented nature of healthcare delivery in these circumstances can result in the provision of services that may be redundant, inconsistent, and even contradictory across different providers, as well as a failure to identify and provide needed services [7]. Furthermore, quality of care in disasters can be adversely impacted by high clinician turnover, which occurs due to changes in personnel availability, closures of permanent healthcare delivery sites, and the presence of short-term medical missions that respond to natural and human-made disasters [8]. Fragmented care also impedes the necessary flow of data to public health authorities to make evidence-based decisions during times of crisis [9,10]. These factors ultimately impact the survival of victims of forced displacement as they seek temporary safety and eventual resettlement, a process which can take several years in some settings [2].

Some of the challenges listed above may be partially addressed through the use of electronic medical records, particularly if adapted to improve communication between providers and public health authorities in disaster settings [11]. However, there is little published experience on the use of electronic medical records between different service providers in resource-limited settings during humanitarian crises, especially as many of these efforts still rely on paper record keeping [12]. In 2011, in response to the preventable loss of life in the aftermath of the earthquake in Haiti, members of our team designed Fast Electronic Medical Records (fEMR), an electronic medical record platform, specifically for use in complex humanitarian disasters and resource-limited settings. Our objective in this study is to review the design and structure of fEMR and determine its uptake, implementation, and usage since its inception.

## Materials and methods

### Platform conceptualization and design

The fEMR system was conceptualized following the January 12, 2010, earthquake in Haiti. At the time, electronic health systems were not readily available for use in settings with severe infrastructure challenges and high turnover in technical and clinical personnel [13]. From 2010–2014, fEMR was developed in an iterative process. The design of fEMR is based on the need to balance certain necessary components of electronic medical records (for example, patient identification, clinical documentation, patient confidentiality) with the unavoidable constraints of disaster medicine, such as the need for efficiency and with high patient and provider turnover. In contrast to most web-based start-ups, which rely on financial investment and privatization, the open source fEMR project was incorporated into curriculum in Computer Science and Engineering degree programs at several academic institutions (namely, Wayne State University, Florida State University, the University of Texas, and California Polytechnic State University). Each term, students contributed to the development of the electronic medical platform and improved on the prior designs. On 2 June 2014, the fEMR system was launched for use in the field. A detailed data structure is available in the Supplement.

The fEMR system was originally deployed on volunteer short term medical trips. In these cases, clinicians, volunteering their time, assist in humanitarian aid efforts in austere settings. Given the brief nature of these initial deployments, the interface is designed so that a clinician can expeditiously complete a clinical encounter with as few clicks as possible and with as little required input necessary to identify each patient. Given the intended patient population, multiple options are available for certain identifiers: for example, a clinician may search for a patient using their name, age, age category (i.e., adult, child, infant, etc.), date of birth, and so on. The majority of the fEMR interface is based on free text to ensure rapid input. The system also incorporates a self-building concept-dictionary to standardize data and reduce variability in clinician input. For example, once a specific diagnosis is entered, the system recalls this the next time a user starts typing related words. If the system saves two different versions of the same word, the end user can delete one by clicking on the small “x” near the word as they are typing. This approach facilitated use by healthcare staff from diverse backgrounds and education whose terminology and colloquialisms might differ, even among speakers of the same language.

### Portability and implementation

**Fig 1:**
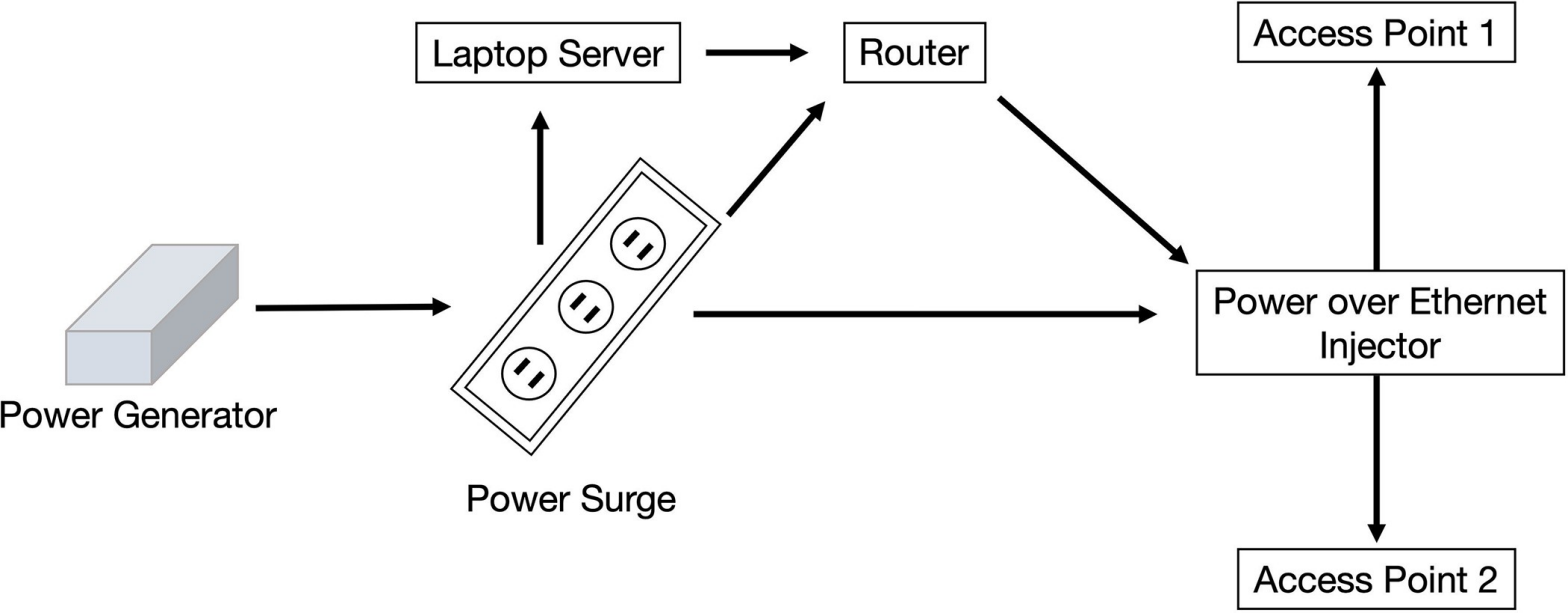
fEMR network setup. Actual equipment may vary slightly depending on the physical layout and geographic location of the clinic. Clinicians connect their own device to the closed intranet connection, then access the fEMR software through Google Chrome.

The original legacy version of the system was designed for areas without access to the internet (i.e., “offline”). In this configuration, the system is equipped with capability to create a closed intranet signal to which clinicians may connect their own device to access the electronic health record software via Google Chrome (Fig 1). The hardware comes in a standard, hard-shell carry-on suitcase that is shipped to the team prior to their deployment. All data are stored on a laptop that acts as a server. To address the lack of electricity, teams either rent a generator or use battery packs.

With growing access to connectivity worldwide, many clinical settings benefited from an online version of the system to suit their needs. To accommodate these settings, an online version of the system was developed, known as fEMR On-Chain. The fEMR On-Chain system provides provable security supported by cryptographically based verification on Amazon’s Quantum Ledger Database, which reduces the risk of data tampering and provides an immutable, cryptographically verifiable log of data changes [14,15]. This proof of all transaction logs allows administrators to analyze and confirm the veracity of any data changes, which is a particularly useful feature given the highly sensitive nature of the patient data collected [15]. Similar to the legacy version, fEMR On-Chain maintains basic demographic and vital information, as well as a complete medical encounter records with spaces for photos, links to imaging, and tests ordered. The decision to move fEMR “on chain” in 2019 was based on feedback from users who worked in clinics based in migrant and refugee camps with stable internet connection. The decline in frequency of deployments to other sites during the first year of the COVID-19 pandemic presented an opportunity for further technical development. Use of fEMR in these settings was continuous (7 days per week), which made it challenging to ship fEMR hardware for data backup and maintenance. Therefore, the online version improved accessibility and usability. There is little required input and an assumption of no billing, so space and time in the system is not devoted to non-essential administrative tasks. The Github repositories for the Legacy version as well as for fEMR On-Chain are available at https://github.com/FEMR.

### Governance, funding, and development

Core fEMR staff and volunteers meet with each end-user after their deployment and discuss feedback. All feedback is reviewed by the founders and then development decisions are made at the beginning of each academic year. Decisions for technical development are based on both degree of importance as well as feasibility of implementation. The leadership team help prioritize technical developments based on the clinical need as directly reported by clinician end-users. Development projects that are beyond the scope of what student developers can complete in one academic year are reserved for software developers who are funded through grant applications and donors. The core team of developers at fEMR, led by the Chief Technical Officer, validates pull requests and lead strategic decisions. Costs for operating fEMR for end-users vary depending on length of deployment and patient load, but it is generally inexpensive to utilize the fEMR system as the organization is largely volunteer-driven and development costs are frequently subsidized by university partnerships.

### Data security and ownership

The fEMR designers and users have identified data security and patient privacy as being a paramount consideration in the deployment of new digital health technologies. Victims of humanitarian crises and forced displacement are a vulnerable population. Compromising data of persons’ identification, whereabouts, and health information can put them at risk of targeting, violence, and extortion. Regulatory standards regarding health information vary across regions and countries. The fEMR team works with clinical response teams to ensure their operations meets the regulations of their host countries in addition to a minimum set of security standards to mitigate potential risks. The technical infrastructure of fEMR is designed with robust security measures including encryption, access controls, and regular security audits, all of which are crucial in preventing data breaches and protecting sensitive patient information. Team fEMR maintains the electronic health data, as well as the technical infrastructure that houses them, in compliance with the US Health Insurance Portability and Accountability Act (HIPAA) and similarly rigorous regulations regarding protected health information mirrored in sites of deployment [16].

Informed consent is not required to be entered into the fEMR system itself, though consent is required in all cases of medical care and is obtained by the clinician providing that care. Most end-users of the system do not have the technical or human capital to maintain the records they authored. Deidentified data may be shared with the end-user as allowable under host-nation parameters, and fEMR designers do not use, examine, or publish any data without acknowledgement from the end-users. Data may also be de-identified and released to certain academic institutions, appropriate government ministries, or UN entities for research purposes, as allowed by the host nations [16–18].

### Usage analysis and feedback

We analyzed routinely collected usage data regarding fEMR implementation from its inception until October 2022. We gathered usage data from all prior fEMR deployments, and estimated the total number of patient encounters, individual patients, and individual clinician users involved in fEMR deployments since its inception. In instances where the On Chain version was used, core technical staff have access to aggregate data regarding fEMR usage (i.e., number of patient encounters, number of unique patients, etc.). In instances where the offline version was used, we estimated the number of patients based on discussions with the end-users as no precise number was readily available. We were unable to retrieve the total number of clinicians who used fEMR during this period. As part of routine operations, after each deployment, end-users had the ability to provide feedback on the system’s functionality. We collected feedback via both open-ended interviews as well as a structured online instrument. In some cases, we had more in-depth meetings with end-users wherein development changes were discussed. We summarized user feedback since the system’s inception to determine its impact and design. We categorized user experiences into one of the following domains: data; hardware; interface; workflow; and miscellaneous.

### Ethics statement and data availability

This study was classified as non-human subjects research by the Mass General Brigham Institutional Review Board. The post-deployment survey instrument, de-identified responses from the software usage surveys, and the Github repositories for the software may be found here: https://github.com/FEMR.

## Results

**Fig 2:**
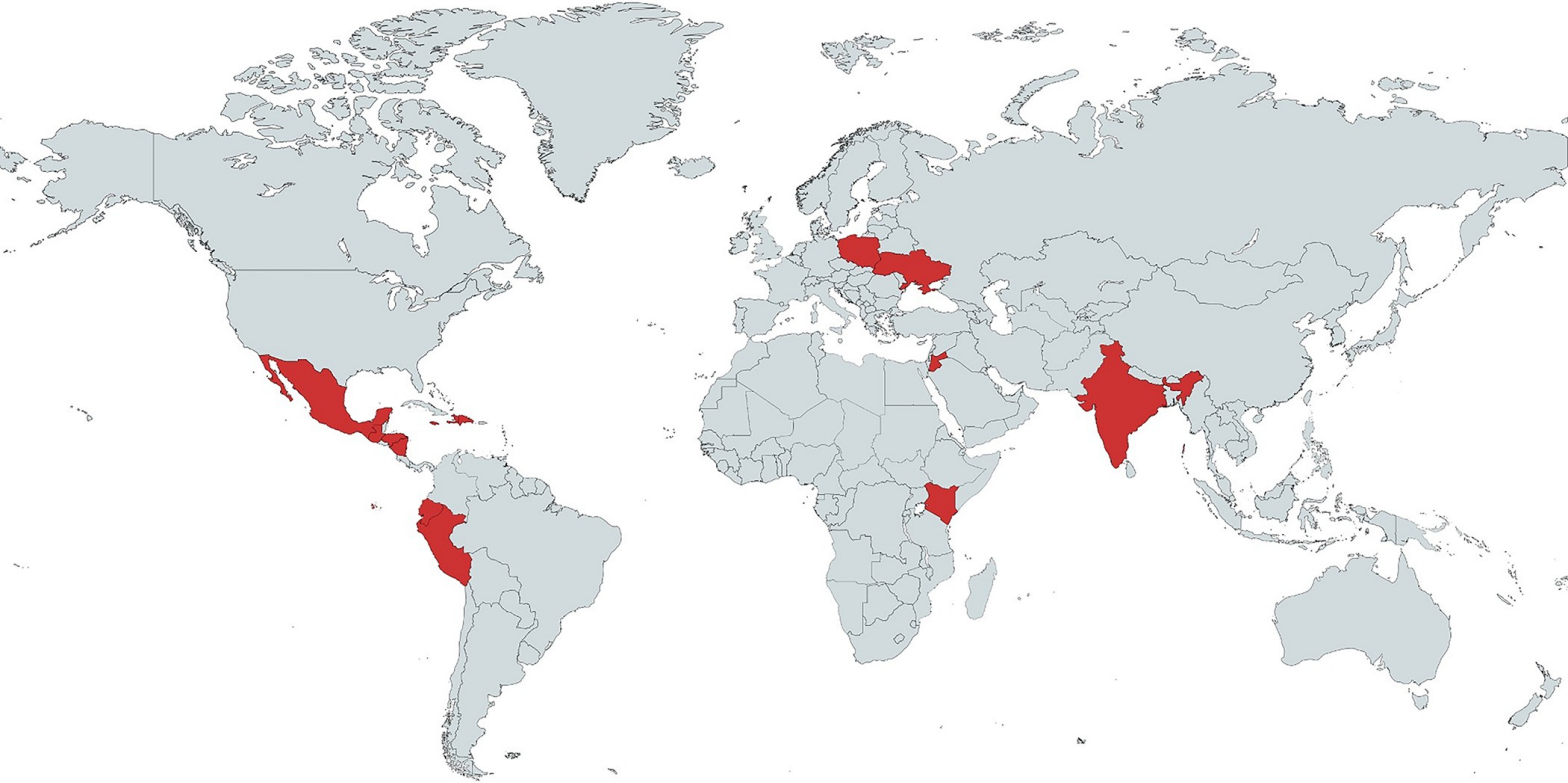
Countries utilizing fEMR during humanitarian deployments, 2014–2022. Map graphic created with MapChart (https://www.mapchart.net). Abbreviations: fEMR = fast Electronic Medical Record.

The fEMR system has been deployed 60 times to 11 different countries by 14 different organizations or institutions since inception (Fig 2). These deployments collectively account for over 37,500 patient encounters with 31,940 distinct patients. The system has been used by teams from nonprofit organizations and academic medical centers from North America (Table 1). The users of the system are all independent of each other and of the core fEMR team. Users learn about fEMR via word-of-mouth or at academic global health conferences. Of these, 58 deployments utilized the legacy version of fEMR, and two have employed fEMR On-Chain. Throughout these deployments, fEMR is fully self-sufficient, but users occasionally bring paper records as a back-up in case they are needed.

**Table 1:** Estimated usage of fEMR (fast electronic medical record), 2014–2022.

Country of Deployment	Number of Deployments	Number of Patients	Number of Encounters	Years	Average Duration
Dominican Republic	9	3,179	4,549	2015–2018	One week for each deployment
Ecuador	4	772	835	2014–2017	One week for each deployment
Guatemala	4	1,661	1,695	2017–2019	One week for each deployment
Haiti	19	4,542	5,254	2014–2019	One week for most deployments
Honduras*	1	500*	500*	2018	One week
India*	1	200*	200*	2015	One week
Jamaica*	2	300*	300*	2019	One week for each deployment
Jordan*	1	150*	150*	2018	One week
Kenya*	1	160*	160*	2020	Two weeks
Mexico	3	17,581*	21,307*	2019–2023	Prolonged deployment lasting >3 years
Nicaragua	4	1,170	1,208	2017, 2018	One week for each deployment
Peru	10	1,557	1,170	2017–2019	Two weeks for each deployment
Ukraine	1	168	173	2022-Present	Intermittently active deployment for >2 years
**Total**	**60**	**31,940**	**37,501**	**2014-Present**	

* The precise number of patient records and clinical encounters could not be determined from deployments which utilized the legacy version of fEMR so estimates were made based on debriefing meetings with clinical teams.

### Deployment settings

The deployment settings evolved from short-term medical missions to a variety of other settings, including care delivery in refugee and migrant encampments, disaster response scenarios, and care delivery in rural or austere settings. For example, fEMR was utilized in 19 separate deployments in Haiti between 2014–2019 accounting for 5,254 different patient encounters. Most of its use in this setting was in short-term primary care clinics in both urban and rural settings. In contrast, use of fEMR in Mexico operations were in long-term clinics aimed at providing primary care and urgent care to migrants, refugees, and asylum seekers seeking entry into the US. The use of fEMR in Mexico included operations at the Northern Mexico border, in the Rio Grande Valley (state of Tamaulipas), and near the Southern Mexico border in the state of Chiapas. Some settings included continuity for the same patients over several years. For example, fEMR was used for an average of 1.43 visits per patient in a series of 9 deployments over three years in the Dominican Republic. The most recent setting of deployment of the fEMR system was in the Poland-Ukrainian border in response to the outbreak of war in Ukraine in February 2022. In this setting, fEMR is used in medical response clinics to refugees fleeing the conflict.

### User feedback

Our analysis of user feedback demonstrated several key themes related to clinician turnover. The simple and intuitive user interface allowed for most clinicians to quickly learn and adapt to the program, despite having a variety of backgrounds and prior experiences with digital health technologies. The ability to confidentially record a unique patient identifier allowed some settings to manage chronic conditions in patients over both one single deployment and multiple recurrent deployments to the same site. Additionally, users assigned value to analyzing clinical records as a source of information for clinical and public health planning. Specifically, users from deployments in the long-term clinics in Mexico noted that personnel and medication management decisions in deployment can be guided by analysis of fEMR records.

Several challenges were described by designers and users in fEMR implementation (Table 2). First, the legacy version of fEMR required that a member of the provider team set up the system at the start of each clinical session and take the system down at the end of each clinical session, a process that typically lasts ten minutes and consumed personnel time. Second, it was challenging for remote technical staff to access the system running ‘offline’ to troubleshoot potential problems. Third, all the data are stored on a laptop server, which limits security, portability, and data feedback to public health authorities and clinical leadership. Establishment of internet connection was necessary for these data to be backed up to a remote server. Moreover, while the reliance on free text entries facilitated use by clinicians in the field, it meant that analysis of fEMR data required additional data cleaning by back-end staff to assign diagnosis codes and ensure consistency in clinical documentation.

**Table 2:** Summary of clinical user feedback on fEMR following deployments.

Domain	User Feedback
Data	Medication data not consistently reported in data fields. Users desired continuity of patient records across different deployments.
Hardware	Difficulty transporting the offline hardware to remote locations. Antenna used in offline hardware is at risk of over-heating in extreme weather.
Interface	Simplification of free text fields. Make prominent the patient’s allergy list in each screen. The system should emphasize abnormal vital signs (using bold or color typeface) when entered in triage.
Workflow	Utilize keyboard shortcuts to expedite workflow.
Miscellaneous	Difficulty in editing patient chart after closing the encounter. Challenges in training on the system as a team prior to deployment. Easy on boarding and efficient learning curve for first-time users.

Because fEMR On-Chain is an online program, technical staff can log on and fix bugs at any time. The data is securely stored in the cloud, and there is no setup or take down necessary. However, if the internet connection is weak, certain sections of the interface may be slow to load, leaving clinicians to enter information in whatever forms are visible to them. Since the original legacy version of fEMR creates its own signal, there are no such connectivity issues. The environments also create unique challenges, regardless of the system that is deployed. There are typically no technical professionals nearby to replace parts or assist with computer problems. Finally, funding challenges were identified. While academic partnerships subsidize development costs, the fEMR systems are deployed to low resource settings, where funding is often difficult or impossible to sustain.

## Discussion

The confluence of human-made events and natural disasters, particularly in the setting of climate change, increases the frequency and consequences of large-scale, complex humanitarian disasters [2]. Healthcare delivery in these settings is met with a number of challenges, including resource limitations, a transient and mobile patient and clinician population, and a breakdown of traditional reporting systems for public health response [9,10]. Implementation of electronic health records in resource-limited settings may help address some of these care needs [12,13]. However, many existing electronic medical record systems are designed for use in high-income healthcare systems and are characterized by complex interfaces and a high learning curve for the user to gain proficiency. In this study, we evaluated the use of fEMR, an electronic record system specifically designed for use in disaster response and resource-limited settings. Two versions of fEMR are compared: first, the legacy version, which does not depend on a continuous internet connection and second, the online (“On-Chain”) version which leverages an internet connection in the clinical setting. Our analysis shows that fEMR On-Chain appears to be more commonly implemented in recurrent and longer-term deployments, likely a result of there being stable internet connectivity in those settings.

Our review of fEMR usage highlighted three broad categories of value provided by an EMR system designed for disaster response settings: clinical care to individual patients, operational efficiency and quality, and public health reporting. For individual patients and providers, an electronic system provides continuity of care. Our analysis of fEMR usage showed that while many short-term deployments did not treat patients in a longitudinal basis, other long-term and recurrent deployments did provide continuity for patients. Like other EMRs that can be rapidly deployed in response settings, fEMR can be useful for the management of chronic conditions across different providers. Future work should analyze the kinetics of clinical documentation entry to better understand the efficiencies and inefficiencies of the system.

A second area of value in EMR implementation is in improving operations for deployment teams. For example, the World Health Organization has a minimum dataset requirement of reporting for emergency medical teams. Use of fEMR has allowed response teams to transition away from collecting these data on paper and instead query reports through fEMR, which improved efficiency and security, based on user feedback. Third, users noted that the ability to query the EMR system for clinical information–albeit rudimentary given the circumstances of the deployments–allowed for clinical collaborations with public health authorities and researchers to better understand population-level health needs. For example, analysis of fEMR data implemented in clinical deployments for migrating people encamped near the US-Mexico border demonstrated the clinical needs of the population during the first year of the COVID-19 pandemic [19]. However, the preponderance of free text data limited the consistency by which data were entered and the efficiency with which these data could be cleaned and analyzed for research purposes.

Digital health technologies are increasingly being implemented in resource-limited settings, including in the context of humanitarian emergencies, and the evidence base supporting digital health interventions in these contexts is growing [20–22]. Electronic health records were implemented during the 2015 Ebola epidemic, though with some logistical delays in deployment [23]. In Turkey, a mobile health application facilitates health-related communication and access to outpatient and urgent medical care for refugees and displaced persons [21,22]. A recent review of digital health technologies in humanitarian settings found numerous published reports of more than 50 different technologies, ranging from community education and engagement technologies to registration and electronic health applications [13]. However, few of these experiences reported the volume and nature of usage and user feedback. Importantly, digital health technologies, including electronic health records, are serving an important role in disease surveillance and as a source of population-level data to guide public health response and research in humanitarian crises [9,11].

There are several ways by which implementation of portable electronic medical records in resource-limited settings may improve clinical and public health outcomes in emergency, humanitarian, and disaster response. First, electronic medical records provide a level of care continuity for people receiving care across different clinicians. Second, electronic medical records can be leveraged to enhance patient safety and quality of care through a variety of mechanisms, including practice reminder alerts and decision support tools [22–25]. Third, electronic medical records can be a source of data for syndromic surveillance and public health monitoring, for example, of infectious pathogens in settings vulnerable to outbreaks of communicable diseases [26]. The fEMR system, like other electronic record systems used in transient medical settings, should eventually transition to a more permanent electronic health record solution, which would require integration to ensure continuity of care and preservation of valuable health information collected during the humanitarian response phase. As more regions and countries develop electronic health records, the interoperability of humanitarian response records and their capacity for integration will become more important.

## Conclusion

In conclusion, we designed fEMR, a novel electronic medical record system for use in humanitarian medical response in resource-limited settings. Over an eight-year period, we successfully deployed this system more than 60 times to reach over 30,000 patients. This low-budget project, which has been based almost entirely on volunteer effort and with input from multidisciplinary clinicians, information technologists, and software engineers, has yielded valuable insights into the provision of clinical care in austere environments. However, the development of fEMR also came with valuable lessons in operations and adapting to a variety of technical capabilities. It is possible that this type of technology, especially the On-Chain format, can become a common tool across WHO and other multilateral emergency medical teams [27]. This, in turn, can facilitate connectivity across non-profit organizations and public health entities who adopt the same tool.

These experiences reflect a variety of clinical settings, including short- and long-term medical responses in resource-limited settings, migrant and asylum-seeker encampments, natural disasters, and conflict zones. By employing an online and offline version of the system, we adapted the use of fEMR for settings with and without reliable internet connectivity. User feedback demonstrated that the system was efficient and easy to learn for clinicians with a background in electronic clinical documentation. Future work should be devoted to efficiently translating clinical data from fEMR into quality measures and public health surveillance.

## Supporting information

S1 Fig Detailed data schematic of fEMR. Footnote to S1 Fig. Schematic shown for legacy fEMR Database Model (December 7, 2021). For a more detailed explanation, see github repository at https://github.com/FEMR.
(TIFF)
