# Supplementary figures and images for: Development and implementation of an electronic health record system for use in humanitarian emergencies, disaster response, and conflict zones
Source: PLOS Glob Public Health. 2025 Jan 29;5(1):e0003124. doi: 10.1371/journal.pgph.0003124 (PMC11778788; doi:10.1371/journal.pgph.0003124)

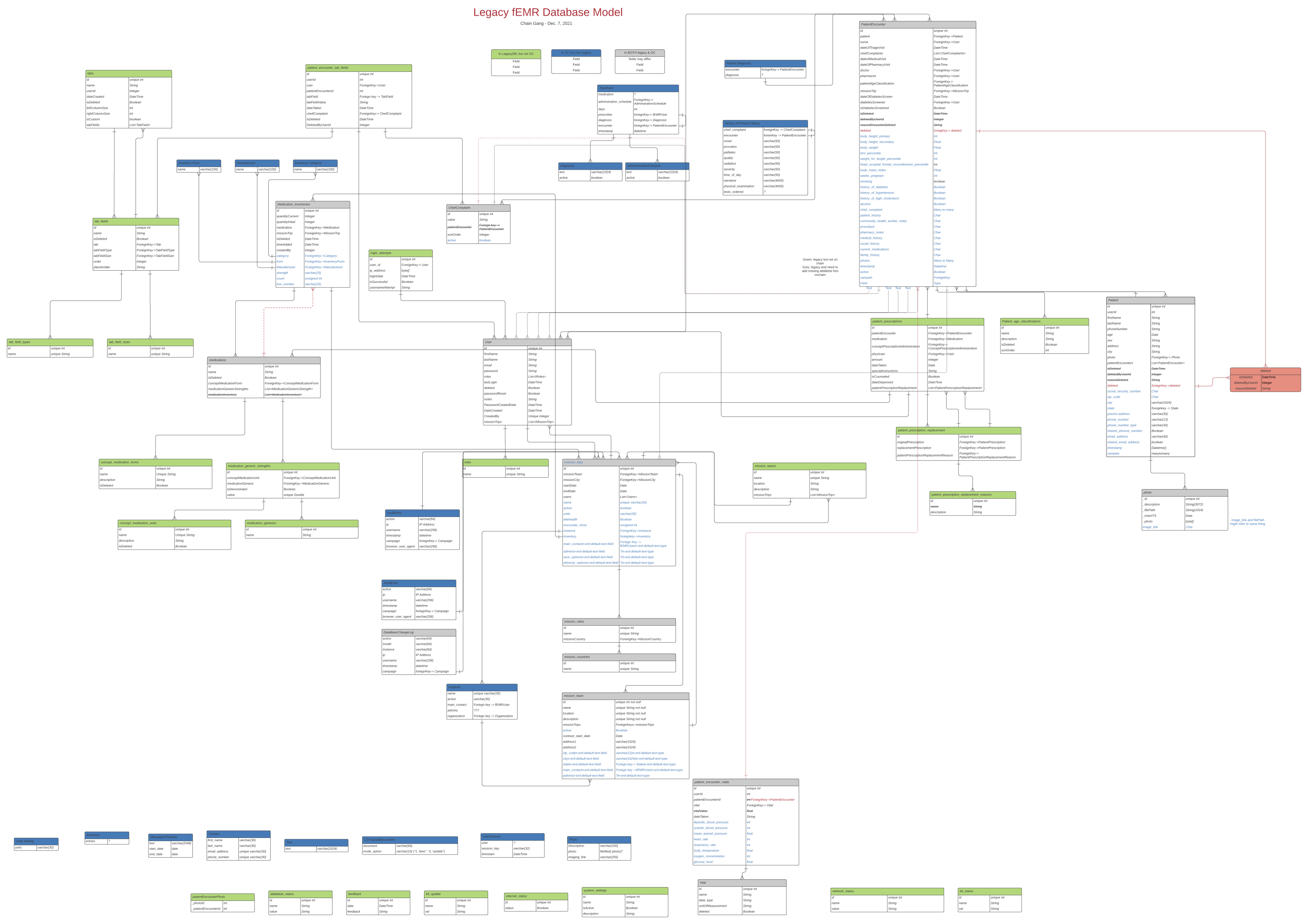

Supplement: S1 Fig — Footnote to S1 Fig. Schematic shown for legacy fEMR Database Model (December 7, 2021). For a more detailed explanation, see github repository at https://github.com/FEMR. (TIFF) [file pgph.0003124.s001.tiff]
